# Safety and Efficacy of a Neonatal Fc Receptor Antagonist in Patients With Anti‐NMDAR Encephalitis

**DOI:** 10.1111/cns.70534

**Published:** 2025-08-11

**Authors:** Zhihong Bian, Han Cai, Haotian Wu, Ping Liu, Yanfang Zuo, Fuhua Peng, Wei Qiu, Zhengqi Lu, Bingjun Zhang

**Affiliations:** ^1^ Department of Neurology The Third Affiliated Hospital of Sun Yat‐Sen University Guangzhou China; ^2^ Department of Neurology Longgang Central Hospital of Shenzhen Shenzhen China

**Keywords:** anti‐NMDAR encephalitis, autoimmune encephalitis, efgartigimod, FcRn receptor, immunotherapy

## Abstract

**Background:**

This retrospective study investigates the safety and effectiveness of efgartigimod in improving clinical outcomes for patients with anti‐NMDAR encephalitis in a real‐world setting.

**Methods:**

We analyzed data from 26 patients diagnosed with anti‐NMDAR encephalitis at the Third Affiliated Hospital of Sun Yat‐sen University between October 2022 and June 2024. The patients were divided into two groups: 13 received efgartigimod treatment, while 13 did not. Clinical outcomes were assessed using the modified Rankin Scale (mRS), the Clinical Assessment Scale in Autoimmune Encephalitis (CASE), and clinical symptoms, along with an analysis of treatment‐emergent adverse events (TEAEs), cerebrospinal fluid (CSF) antibody titers, and blood serum IgG levels.

**Results:**

Efgartigimod treatment was associated with significant clinical improvement, as indicated by greater reductions in mRS and CASE scores at discharge and follow‐up compared to the control group. At follow‐up, 84.6% of patients in the efgartigimod group achieved an mRS score of ≤ 2, compared to 46.2% in the control group. Additionally, patients receiving Efgartigimod exhibited a notable reduction in CSF anti‐NMDAR antibody titers and serum IgG levels. The most common TEAEs were mild to moderate infections, with no significant safety concerns identified.

**Conclusion:**

In this exploratory study, efgartigimod demonstrated a favorable safety profile for patients with anti‐NMDAR encephalitis and appeared to facilitate the recovery of clinical symptoms and neurological function. However, further prospective randomized studies with larger patient cohorts are necessary to confirm the safety and efficacy of efgartigimod.

## Graphical abstract

Efgartigimod reduces IgG recycling and promotes the degradation of IgG and pathogenic autoantibodies. Efgartigimod treatment for anti‐NMDA receptor encephalitis was associated with no apparent adverse effects and accelerated improvements in neurological dysfunction and clinical symptoms, particularly cognitive function. Efgartigimod represents a promising novel therapeutic option for anti‐NMDAR encephalitis.

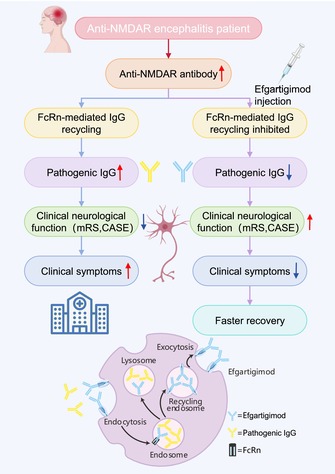

**Abbreviations**
AE: adverse event
CASE: Clinical Assessment Scale for Autoimmune Encephalitis
CSF: cerebrospinal fluid
FcRn: neonatal Fc receptor
ICU: intensive care unit
IgG: immunoglobulin G
IVIG: intravenous immunoglobulin
mRS: modified Rankin Scale
NEOS: anti‐NMDAR encephalitis 1‐year functional status
NMDAR: N‐methyl‐D‐aspartate receptor
SD: standard deviation
TEAE: treatment‐emergent adverse event

## 1 Introduction

Anti‐N‐methyl‐D‐aspartate receptor (NMDAR) encephalitis is a severe autoimmune disorder marked by autoantibodies targeting NMDA receptors, resulting in significant neurological and psychiatric symptoms [1]. This condition typically presents with a rapid onset, including psychiatric disturbances, cognitive impairment, seizures, movement disorders, and autonomic dysfunction [2]. It is rare, with an annual incidence of 1.5 cases per million people, and is more prevalent in females [3].

Initial treatment for anti‐NMDAR encephalitis generally includes corticosteroids, intravenous immunoglobulin (IVIG), and plasma exchange (including immunoadsorption) to mitigate the inflammatory response and reduce pathogenic antibody levels. Second‐line therapies primarily involve rituximab and cyclophosphamide [4]. Approximately 80% of patients may achieve near‐full recovery through immunotherapy; however, 20%–30% of patients remain refractory [3]. Moreover, the current treatment options have several limitations: plasma exchange and immunoadsorption are highly invasive and costly procedures; prolonged corticosteroid therapy predisposes patients to metabolic complications; IVIG requires repeated high‐dose infusions; and rituximab or cyclophosphamide increase the risk of serious infections [5, 6, 7]. Considering these shortcomings, antagonists targeting the neonatal Fc receptor (FcRn) offer a novel therapeutic approach. They accelerate IgG catabolism in vivo, thereby removing pathogenic antibodies without the need for plasma exchange.

Efgartigimod, an innovative FcRn antagonist, has emerged as a promising therapeutic option for autoimmune diseases. By binding to the neonatal Fc receptor, efgartigimod inhibits its interaction with immunoglobulin G (IgG), reducing IgG recycling and promoting the degradation of IgG and pathogenic autoantibodies, while preserving the levels of other immunoglobulins and albumin [8, 9, 10]. This mechanism allows for the safe breakdown of IgG, potentially lowering NMDAR antibody levels [10]. Administered as an IV infusion, efgartigimod is a less invasive alternative to plasma exchange or immunoadsorption, potentially improving patient compliance. Importantly, it does not compromise immune responses and is associated with few, generally mild, and transient side effects; significant toxicity has not been observed in prior studies [9, 11, 12]. In the field of neuroimmunology, efgartigimod was approved in 2021 by regulatory agencies in the United States, the European Union, and Japan for the treatment of generalized myasthenia gravis (gMG), having significantly improved MG‐ADL and QMG scores in the phase III ADAPT trial [13, 14]. Subsequently, the phase II/III ADHERE study demonstrated that efgartigimod markedly reduced the risk of relapse in chronic inflammatory demyelinating polyneuropathy (CIDP) [15]. Encouraging clinical results have also been reported in other IgG‐mediated disorders, including primary immune thrombocytopenia and pemphigus [16, 17]. Although FcRn antagonists have been shown to be effective in a range of neuroimmunological disorders, evidence for their use in autoimmune encephalitis remains limited to a few small case series and single‐patient reports; comprehensive clinical studies are still lacking [18, 19].

Assessing the effectiveness and safety of efgartigimod for anti‐NMDAR encephalitis is essential. This study aimed to investigate these parameters and expand the current understanding of treatment options for this condition. We performed a retrospective analysis of treatment‐emergent adverse events (TEAEs), clinical outcomes, initial symptoms, and laboratory results in patients with anti‐NMDAR encephalitis treated with efgartigimod, comparing them with those who received alternative treatments.

## 2 Methods

### 2.1 Study Design and Patients

Patients diagnosed with definite anti‐NMDAR encephalitis according to the consensus criteria were included in this retrospective study [6]. The efgartigimod treatment regimen consisted of 800 mg per dose administered weekly over a two‐week period. An indirect fluorescence assay (EUROIMMUN, Medizinische Labordiagnostika, Lubeck, Germany) was utilized to detect IgG antibodies against the NMDAR [20]. This study received approval from the Institutional Review Board of The Third Affiliated Hospital of Sun Yat‐Sen University, China. All participants or their legal representatives provided written informed consent. All eligible patients admitted within the study window were enrolled consecutively.

### 2.2 Inclusion and Exclusion Criteria

The inclusion criteria were as follows: patients aged ≥ 14 years diagnosed with definite anti‐NMDAR encephalitis according to the consensus criteria, which included: (1) rapid onset (within 3 months) of one or more of the six major symptom groups: abnormal behavior or cognitive dysfunction, speech dysfunction, seizures, movement disorders, decreased level of consciousness, and autonomic dysfunction or central hypoventilation; (2) presence of IgG anti‐GluN1 antibodies; (3) a modified Rankin Scale (mRS) score of ≥ 2 at baseline; and (4) no use of IVIG or plasma exchange/immunoadsorption within 7± days before baseline for the efgartigimod treatment group.

The exclusion criteria were as follows: (1) missing key clinical parameters or loss to follow‐up; (2) total IgG level of ≤ 6 g/L at admission; and (3) severe underlying conditions such as heart failure, arrhythmias, or coagulation disorders, which the investigator deemed ineligible for the study [6].

### 2.3 Analysis of Clinical, Laboratory, and Immunotherapy Profiles

Data were extracted for the following parameters from clinical records: gender, age, clinical manifestations, time from onset to hospitalization, length of hospital stay, length of follow‐up, plasma and cerebrospinal fluid (CSF) test results, presence of tumors, NMDA receptor antibody titers in CSF and plasma, treatment‐related adverse events, clinical outcomes, and details of any immunotherapy administered. First‐line immunotherapy included corticosteroids, plasma exchange/immunoadsorption, and IVIG, while second‐line therapy encompassed efgartigimod and other immunotherapies. Neurological functional status was assessed using the mRS (scores ranging from 0 to 6) and the Clinical Assessment Scale for Autoimmune Encephalitis (CASE; scores ranging from 0 to 27) at admission, discharge, and follow‐up, defined as readmission within 1–3 months post‐discharge.

### 2.4 Assessment of Treatment Efficacy

To assess the efficacy of immunotherapies, the primary outcome was the change in the modified Rankin Scale (mRS) score from baseline to follow‐up. The secondary outcomes included changes in the Clinical Assessment Scale in Autoimmune Encephalitis (CASE) score, clinical symptoms, the results of CSF and Blood Serum Analysis, and safety outcome. A favorable prognosis is defined as an mRS score of 0 to 2 at follow‐up.

### 2.5 Assessment of Adverse Effects

Adverse events (AEs) that emerged or worsened after the initiation of treatment were classified as treatment‐emergent adverse events (TEAEs). For each AE, the investigator determined severity (mild, moderate, or severe) and causality (unrelated, unlikely, possibly, probably, or certainly related). TEAEs were considered related to efgartigimod if classified as “possibly,” “probably,” or “certainly” related.

### 2.6 Statistical Analysis

Statistical analyses were performed using GraphPad Prism (version 9.0, GraphPad Software Inc., San Diego, CA, USA), with results expressed as mean ± standard deviation (SD) or median (interquartile range). Normality was assessed using the D'Agostino–Pearson omnibus test. Continuous variables among three or more groups were compared using the Kruskal–Wallis test, followed by Dunn's multiple comparisons test. The Mann–Whitney test was used for comparisons between two groups. For paired data, the Wilcoxon signed‐rank test evaluated differences between related samples. The candidate variables with *p* < 0.2 in univariate testing were included in the multivariate logistic regression to determine the independent predictors of favorable prognosis; adjusted odds ratios (aOR) with 95% CIs are reported. Ordinal variables were compared using Fisher's exact test, with a *p*‐value of < 0.05 considered statistically significant.

## 3 Results

### 3.1 Demographics and Clinical Characteristics

This retrospective study enrolled 26 patients diagnosed with anti‐NMDAR encephalitis who were admitted to the Department of Neurology at the Third Affiliated Hospital of Sun Yat‐Sen University from October 2022 to June 2024.

**FIGURE 1:**
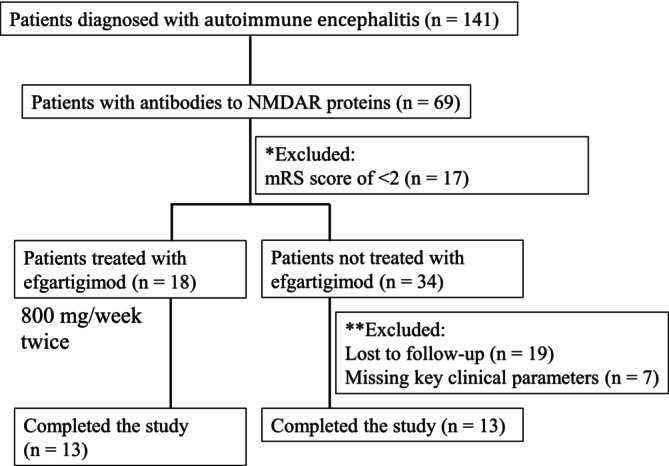
Study population profile. The number of patients in the efgartigimod and control treatment arms is depicted. *Patients were excluded because the mRS score was not fulfilling the criteria for inclusion (*n* = 17). **Patients were excluded because they were lost to follow‐up (*n* = 19) or had missing information on key clinical parameters (*n* = 7).

We identified 141 patients diagnosed with autoimmune encephalitis through the hospital's medical record system. Among them, 69 were confirmed to have NMDA receptor encephalitis (Figure 1). According to our inclusion and exclusion criteria, we excluded patients with an mRS score of less than 2 (*n* = 17), those missing key clinical information (*n* = 7), and those lost to follow‐up (*n* = 19). Ultimately, 26 patients met the criteria, with 13 receiving efgartigimod treatment (age: 23.9 ± 11.3 years, female ratio 8:7, *p* = 0.59) and 13 not receiving it (age: 26.9 ± 13.0 years, female ratio 6:9, *p* = 0.70) (Table 1). The median mRS score and the median CASE score indicate the severity of anti‐NMDAR encephalitis. Multivariable analysis confirmed that age (OR 0.98, 95% CI 0.91–1.1, *p* = 0.645), gender (OR 1.20, 95% CI 0.18–7.89, *p* = 0.849), ICU admission at presentation (OR 4.09, 95% CI 0.24–108.30, *p* = 0.351), time from onset to medication (OR 0.96, 95% CI 0.79–1.17, *p* = 0.689), baseline CASE score (OR 0.85 95% CI 0.58–1.20, *p* = 0.368) and baseline mRS score (OR 1.43, 95% CI 0.25–9.07, *p* = 0.69) were not independently associated with receiving efgartigimod (Table S1). Among all patients, one (3.8%) had an ovarian teratoma, one (3.8%) had a thyroid tumor, and no other tumors were identified. Two patients (7.6%) required mechanical ventilation, and 11 (42.3%) were admitted to the ICU. Abnormal MRI findings (50.0%) and abnormal EEG findings (76.9%) in total patients. All patients tested positive for CSF anti‐NMDAR antibodies, and 78.3% of total had positive serum anti‐NMDAR antibodies. All patients presented with abnormal CSF white blood cell (WBC) counts, and 6 patients (25.0%) had elevated CSF protein levels. No significant differences in baseline data, clinical symptoms, or CSF examination results were observed between the efgartigimod and control groups at admission (*p* > 0.05; Table 1).

**TABLE 1:** Characterization of the NMDAR patient cohort.

	Efgartigimod (*n* = 13)	Control (*n* = 13)	*p*
Age, years; mean ± SD	23.9 ± 11.3	26.9 ± 13.0	0.59
Male/Female	8: 5	6: 7	0.70
CASE on admission; median (IQR)	16 (8)	17 (10)	0.59
mRS on admission; median (IQR)	5 (1.5)	5 (1)	0.76
Time from onset to medication; median (IQR)	5 (4.5)	8 (7.5)	0.77
Symptoms; *n* (%)			
Seizures	9 (69.2)	10 (76.9)	> 0.99
Psychiatric behavioral abnormalities	12 (92.3)	10 (76.9)	0.59
Cognitive impairment	12 (92.3)	12 (92.3)	> 0.99
Speech impairment	11 (84.6)	11 (84.6)	> 0.99
Movement disorder or involuntary movements	9 (69.2)	10 (76.9)	> 0.99
Decreased level of consciousness	8 (61.5)	9 (69.2)	> 0.99
Autonomic dysfunction or central hypoventilation	10 (76.9)	9 (69.2)	> 0.99
Ventilator support; *n* (%)	1 (7.7)	1 (7.7)	> 0.99
Length of hospital stay, days; median (IQR)	19 (11.5)	24 (11.5)	0.32
Length of follow‐up, days; median (IQR)	50 (21)	40 (31.5)	0.53
ICU admission; *n* (%)	5 (38.5)	6 (46.2)	> 0.99
Abnomal MRI on admission; *n* (%)	6 (46.2)	7 (53.9)	> 0.99
Abnomal EEG on admission; *n* (%)	10 (83.3)	10 (90.9)	> 0.99
Tumor; *n* (%)	0	2 (15.4)	0.48
Immunotherapy; *n* (%)			
Steroids	13 (100.0)	13 (100.0)	> 0.99
Intravenous Immunoglobulin	7 (53.9)	11 (84.6)	0.20
Immunoadsorption	0	2 (15.4)	0.48
Rituximab	3 (23.1)	6 (46.2)	0.41
Ofatumumab	2 (15.4)	0	0.48
Azathioprine	1 (7.7)	0	> 0.99
Mycophenolate Mofetil	4 (30.8)	4 (30.8)	> 0.99
CSF NMDAR antibody positive	13 (100.0)	13 (100.0)	> 0.99
Serum NMDAR antibody positive	11 (84.6)	7 (70.0)	0.33
CSF protein on admission (> 0.45 g/L)	2 (16.7)	4 (33.3)	0.64
CSF WBC count on admission (> 5 cells/uL)	12 (100.0)	12 (100.0)	> 0.99

Abbreviations: CASE, Cognitive Assessment Scale for Encephalitis; CSF, Cerebrospinal fluid; EEG, Electroencephalogram; ICU, Intensive Care Unit; MRI, Magnetic Resonance Imaging; mRS, Modified Rankin Scale; NMDAR, N‐methyl‐D‐aspartate receptor; SD, Standard Deviation; WBC, White Blood Cell.

### 3.2 Immunotherapy

Both the efgartigimod and control groups received first‐line immunotherapy, with all patients undergoing corticosteroid treatment (Table 1). Additionally, patients received IVIG. Immunoadsorption therapy showed no differences between the two groups (*p* > 0.05); no patients received plasma exchange. The most common first‐line therapy combination was corticosteroids and IVIG (*n* = 18, 69.2%), while few patients opted for plasma exchange or immunoadsorption therapy (*n* = 2, 7.7%).

In the second‐line therapy, efgartigimod was the most commonly used agent, followed by rituximab (*n* = 9, 34.6%). All patients were given gradually tapered oral steroids for long‐term maintenance. Detailed pre‐efgartigimod immunotherapy exposure is summarized in Table S2.

### 3.3 Primary Outcome of Efficacy

#### 3.3.1 CASE Scores Improvement

**FIGURE 2:**
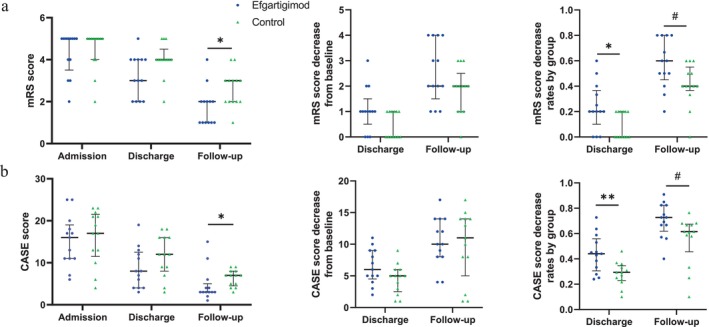
Neurological function changes in NMDAR patients. The modified Rankin Scale (mRS) and the Clinical Assessment Scale for Autoimmune Encephalitis (CASE) scales were used to assess the patients' neurological recovery. Values are presented as median ± interquartile range. (a) Distribution and changes in CASE scores at admission, discharge, and follow‐up for patients in the efgartigimod treatment group and the control group. (b) Distribution and changes in mRS scores at admission, discharge, and follow‐up in the efgartigimod treatment group and the control group (*p < 0.05, **p < 0.01, and ^#^ *p* < 0.05).

Clinical neurological function was assessed using the mRS score at three time points: admission, discharge, and follow‐up (Figure 2) as the primary outcome. The mRS score showed significant reductions post‐treatment in both groups (Figure 2a). At follow‐up, the mRS (*p* = 0.021) was significantly lower in the efgartigimod group (media*n* = 2) than in the control group (median = 3). Notably, 84.6% (*n* = 11) of patients in the efgartigimod group achieved an mRS score of 2 or less at follow‐up, compared with 46.2% (*n* = 6) in the control group (Figure 2a). The reductions in mRS score at discharge and follow‐up were not significantly different between the two groups (*p* > 0.05). However, the rate of decrease in mRS score was significantly greater in the efgartigimod group compared to the control group at both discharge (efgartigimod, median = 0.2; control, median = 0; *p* = 0.032) and follow‐up (efgartigimod, median = 0.6; control, median = 0.4; *p* = 0.037). The average reduction rate in mRS score at follow‐up for the efgartigimod group was 57.7%. Furthermore, the average duration of hospitalization was shorter for the efgartigimod group (22.5 ± 10.9 days) than the control group (26.5 ± 16.0 days), although this difference was not statistically significant (Table 1).

A logistic‐regression analysis was conducted to assess the potential influences on some important clinical factors and therapy, as measured by whether mRS ≤ 2 at follow‐up. The interval between symptom onset and the first course of immunotherapy (OR 0.80; 95% CI 0.60–1.00, *p* = 0.064), treatment with efgartigimod (OR 6.35, 95% CI 0.77–102.80, *p* = 0.116), concomitant use of IVIG (OR 0.24, 95% CI 0.01–3.37, *p* = 0.336), or rituximab (OR 0.35, 95% CI 0.03–2.99, *p* = 0.341) were not independently associated with good outcome (Table 2).

**TABLE 2:** Univariate and multivariate logistic regression analysis of the outcome for mRS ≤ 2.

Parameter	Univariate	Multivariate	
	*p*	OR (95% CI)	*p*
Time from onset to medication	0.086	0.80 (0.60–1.00)	0.064
Duration of illness	0.268	—	—
Efgartigimod	0.050	6.35 (0.77–102.80)	0.116
Intravenous immunoglobulin	0.141	0.24 (0.01–3.37)	0.336
Rituximab	0.112	0.35 (0.03 to 2.99)	0.341
Immunoadsorption	0.639	—	—

### 3.4 Secondary Outcomes of Efficacy

#### 3.4.1 CASE Scores Improvement

Disease severity was further quantified with the CASE score at admission, discharge, and follow‐up (Figure 2b). At follow‐up, the efgartigimod group showed a markedly decreased CASE score compared to the control group (efgartigimod, median = 3; control, median = 7; *p* = 0.015). The rate of decrease in CASE score also suggested a significant improvement after being treated with efgartigimod at both discharge (efgartigimod, median = 0.44; control, median = 0.29; *p* = 0.007) and follow‐up (efgartigimod, median = 0.73; control, median = 0.62; *p* = 0.028) (Figure 2b). This faster neurological recovery supported the findings obtained with the mRS score shown.

### 3.5 Time Course of Clinical Symptom Evolution

**FIGURE 3:**
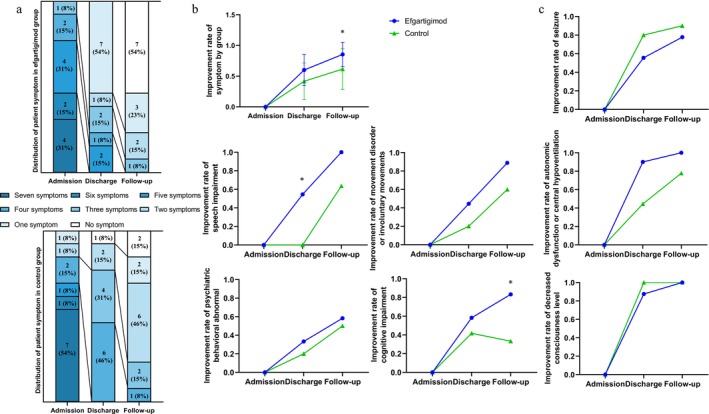
Time course of clinical symptoms' evolution in NMDAR patients. (a) Proportions of patients with anti‐N‐methyl‐D‐aspartate receptor (NMDAR) with no to seven major symptoms (i.e., seizures, psychiatric behavioral abnormalities, cognitive impairment, speech impairment, movement disorders or involuntary movements, decreased level of consciousness, and autonomic dysfunction or central hypoventilation) at admission, discharge, and follow‐up. (b) Changes in the improvement rate of clinical symptoms exhibited by the patient at admission, discharge, and follow‐up. Values are presented as mean ± SD. (c) Changes in the improvement rate of patients with the indicated symptoms at admission, discharge, and follow‐up (*p < 0.05 and ^#^ *p* < 0.05).

Table 1 summarizes clinical symptoms at admission, while Figure 3 illustrates changes in these symptoms at discharge and follow‐up across different treatments. At admission, all patients exhibited the following symptoms: seizures (*n* = 19, 73.1%), psychiatric behavioral abnormalities (*n* = 22, 84.6%), cognitive impairment (*n* = 24, 92.3%), speech impairment (*n* = 22, 84.6%), movement disorders or involuntary movements (*n* = 19, 73.1%), decreased level of consciousness (*n* = 17, 65.4%), and autonomic dysfunction or central hypoventilation (*n* = 19, 73.1%) (Figure 3a). After immunotherapy, both groups demonstrated significant improvements in clinical symptoms, with the efgartigimod group showing a greater reduction rate in symptom numbers at discharge (efgartigimod, median = 0.18; control, median = 0.55; *p* = 0.013) and follow‐up (efgartigimod, median = 0.2; control, median = 0; *p* = 0.032) compared to the control group (Figure 3b). There were no significant differences in the improvement rate of experiencing seizures, movement disorders, autonomic dysfunction, psychiatric behavioral abnormalities, or decreased levels of consciousness between the two groups at discharge and follow‐up (Figure 3c). However, the improvement rate of speech impairment significantly decreased in the efgartigimod group at discharge (*p* = 0.041), though not at follow‐up. Additionally, the improvement rate of cognitive impairment increased in the control group at follow‐up, whereas fewer patients in the efgartigimod group experienced cognitive impairment at follow‐up compared to the control group (*p* = 0.041) (Figure 3c).

### 3.6 CSF and Blood Serum Analyses

**FIGURE 4:**
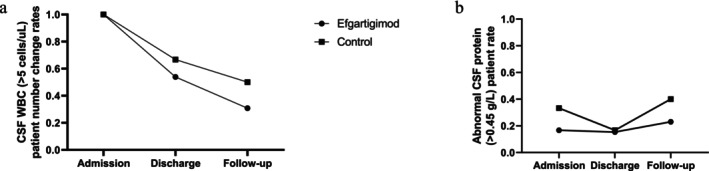
CBF protein and WBC changes in NMDAR patients. (a) Number of patients with anti‐N‐methyl‐D‐aspartate receptor (NMDAR) with abnormal cerebrospinal fluid (CSF) white blood cell (WBC) counts (> 5 cells/uL) at admission, discharge, and follow‐up. (b) Number of NMDAR patients with abnormal CSF protein levels (> 0.45 g/L) at admission, discharge, and follow‐up.

CSF analysis revealed a continuous decrease in the number of patients with abnormal CSF WBC counts (> 5 cells/μL) following treatment in both the efgartigimod group (*n* = 7, 53.8%) and control group (*n* = 8, 66.7%; *p* > 0.05). However, at follow‐up, 4 patients (30.8%) in the efgartigimod group and 5 patients (50%) in the control group continued to exhibit abnormal CSF WBC counts (*p* > 0.05; Figure 4a). The trend in abnormal CSF protein levels (> 0.45 g/L) indicated no significant correlation with treatment progress in either group, as only 25% (*n* = 6) of patients presented abnormal CSF protein levels post‐treatment (Table 1). Notably, the proportion of patients with abnormal CSF protein levels increased at follow‐up in both the efgartigimod group (*n* = 3, 23.1%) and control group (*n* = 4, 40.0%) compared with those at admission (Figure 4b). No significant differences were observed between the two groups at either discharge or follow‐up (*p* > 0.05).

**FIGURE 5:**
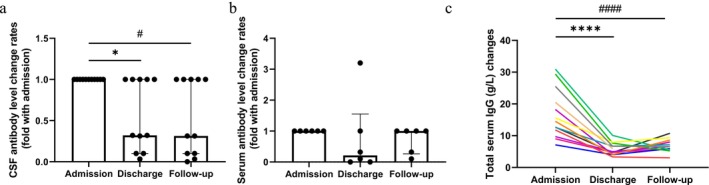
Anti‐NMDAR antibodies and IgG changes after efgartigimod treatment. (a) Changes in the CSF anti‐NMDAR antibodies level in the efgartigimod group. Values are presented as median ± interquartile range (*p < 0.05 and ^#^ *p* < 0.05). (b) Changes in the serum anti‐NMDAR antibody titers in the efgartigimod group. Values are presented as median ± interquartile range. (c) Changes in the serum total immunoglobulin G (IgG) levels in the efgartigimod group (****p < 0.0001 and ^####^ *p* < 0.0001).

In the efgartigimod group, CSF anti‐NMDAR antibody level change rates significantly decreased at discharge (median = 0.32; *p* = 0.031) and follow‐up compared to admission (median = 0.31; *p* = 0.031), with no significant differences between discharge and follow‐up (*p* > 0.05; Figure 5a). Blood IgG levels in the efgartigimod group reached their lowest values at discharge, followed by a slight rebound at follow‐up. This pattern contrasted with the improvement in clinical symptoms observed at follow‐up compared to discharge; however, no significant differences were noted in blood IgG levels (*p* > 0.05; Figure 5b).

### 3.7 Safety Outcomes in Patients

No deaths or severe treatment‐emergent adverse events (TEAEs) that necessitated treatment discontinuation were reported in the study (Table 3). Adverse reactions, such as headaches or allergies, were absent in both groups during treatment. The most common TEAE following efgartigimod treatment was moderate new‐onset infections (*n* = 3, 23.1%), which included pneumonia (*n* = 1) and urinary tract infections (*n* = 2), likely related to efgartigimod. One patient in the control group also experienced moderate new‐onset pneumonia (*p* > 0.99). In contrast, three patients in the control group exhibited moderate elevations in inflammatory markers (PCT or IL‐6), while only one patient in the efgartigimod group showed similar elevations post‐treatment (*p* > 0.99). Both groups experienced one case of moderate white blood cell (WBC) count reduction, with the reduction in the efgartigimod group likely associated with the treatment. Both patients recovered with medical management (*p* > 0.99). Two patients developed moderate fever after efgartigimod treatment, which was deemed possibly related to the drug; however, two cases of moderate fever were also observed in the control group (*p* > 0.99). Other TEAEs possibly related to efgartigimod, including reduced total lymphocyte count, decreased B lymphocytes, and increased neutrophil count, were classified as mild. Overall, there were no significant differences in TEAEs between the two groups (*p* > 0.05).

**TABLE 3:** Safety outcomes emerging from treatment in all patients (overall reported patients > 1).

TEAE/patient count; *n* (%)	Efgartigimod (*n* = 13)	Control (*n* = 13)	*p*
Fever	2 (15.4)	2 (15.4)	> 0.99
Inflammatory markers elevated	2 (15.4)	3 (23.1)	> 0.99
Infections	3 (23.1)	1 (7.7)	> 0.99
Abnormal differential WBC counts	1 (7.7)	1 (7.7)	> 0.99
Total lymphocyte count decrease	2 (15.4)	0	0.48
Monocyte count decrease	1 (7.7)	2 (15.4)	> 0.99
Neutrophil count increase	1 (7.7)	1 (7.7)	> 0.99

Abbreviation: TEAE, treatment‐emergent adverse event.

## 4 Discussion

Our findings indicate that efgartigimod is effective in treating NMDAR encephalitis. Most TEAEs were mild or moderate, with no safety concerns noted compared with the control group. Patients receiving efgartigimod showed more significant improvements in the mRS scores at discharge and follow‐up than those who did not receive the treatment. This study represents the first clinical investigation of FcRn antagonism in treating NMDA receptor encephalitis.

First‐line treatments for anti‐NMDAR encephalitis, such as plasma exchange and immunoadsorption, effectively remove pathogenic IgG. Their utility has been demonstrated in various studies, which show that these methods directly clear pathogenic IgG antibodies during the early stages of the disease [21, 22]. However, plasma exchange and immunoadsorption have limitations, including invasiveness, high costs, and restrictions in patients with coagulation disorders, hypotension, or liver dysfunction, factors that can reduce acceptance among patients and families. The Anti‐NMDAR Encephalitis One‐Year Functional Status (NEOS) score indicates that clinical symptom improvement correlates with better outcomes [23, 24]. Despite first‐line immunotherapy, many patients with NMDAR encephalitis continue to experience severe symptoms, necessitating second‐line treatments [25]. Additionally, plasma exchange and immunoadsorption can lower the blood concentrations of other medications, potentially affecting overall therapeutic efficacy [26]. In contrast, FcRn antagonism, such as efgartigimod, presents a less invasive alternative with a more specific mechanism of action and minimal impact on other medications. Efgartigimod blocks the neonatal Fc receptor (FcRn) inside acidic endosomes. Normally, FcRn rescues IgG molecules from degradation and sends them back into the blood. When efgartigimod binds FcRn, this “recycling” step stops, so both total IgG and disease‐causing IgG are sent to lysosomes and broken down [10]. This mechanism is different from IVIg, which mainly shifts immune signaling through Fc‐dependent pathways and only briefly competes with FcRn without speeding up IgG breakdown. It also differs from corticosteroids, which reduce antibody levels indirectly by stopping immune cells. Once circulating IgG falls, a new gradient pulls pathogenic antibodies out of the CSF and back into the blood—even though efgartigimod never crosses the blood–brain barrier. That matches our results, where serum IgG and CSF anti‐GluN1 antibody levels fell together. Animal studies also follow this idea: in a mouse model of autoimmune encephalitis, FcRn inhibition lowered brain‐bound IgG by more than 70% and improved survival within 48 h [27]. These may explain why patients in the efgartigimod group recovered faster than those who received only steroids or IVIg. A previous study demonstrated the safety and effectiveness of administering efgartigimod at 20 mg/kg weekly for treating myasthenia crisis [28]. Given the acute onset and severe early symptoms of NMDAR encephalitis, particularly in younger patients, we administered 800 mg per dose, twice a week, equating to 20 mg/kg for a 40 kg patient, as there are currently no established dosage recommendations for efgartigimod in autoimmune encephalitis.

We compared the number of patients with an mRS score of ≤ 2 at follow‐up with data from previous studies. In a large historical cohort treated with corticosteroids alone, only about 35% of patients achieved an mRS ≤ 2 by 3 months [29], whereas the combination of steroids + IVIg improved this proportion to about 50% [3]. In a clinical study involving 111 patients with NMDA receptor encephalitis receiving standard first‐ and second‐line treatments (including rituximab), approximately 51% had an mRS score of ≤ 2, with approximately 15% showing a reduction in mRS score from > 2 to ≤ 2 after 2 months of immunotherapy [30, 31]. This result is consistent with the control group in our study. Another clinical study involving 10 patients treated with immunoadsorption therapy found that approximately 70% had an mRS score of ≤ 2 at follow‐up after 3–6 months, all of whom had an mRS score > 2 upon admission [31]. In our study, approximately 85% of patients treated with efgartigimod achieved an mRS score of ≤ 2, with nearly 54% improving from > 2 to ≤ 2. This suggests that efgartigimod may provide better improvements in mRS scores compared to traditional treatments, demonstrating similar efficacy to immunoadsorption in severe NMDA receptor encephalitis. A recent German case series (*n* = 8) described rapid mRS improvement after efgartigimod rescue in steroid‐refractory anti‐LGI1 encephalitis [32]. An ongoing phase 2, open‐label trial in Japan is evaluating efgartigimod as an add‐on to steroids/IVIg in various antibody‐positive encephalitis, which suggested efgartigimod is able to reduce CSF antibody titres [19]. These also point to a broader utility of FcRn antagonism across autoimmune encephalitis. We utilized both the mRS and Clinical Assessment Scale for Autoimmune Encephalitis (CASE) to evaluate patient condition changes. CASE is a novel scale for rating severity in autoimmune encephalitis syndrome [33]. Our results show that trends in CASE and mRS score changes were similar, with the efgartigimod group demonstrating more significant improvement at discharge and follow‐up, highlighting its efficacy in treating NMDA receptor encephalitis. We observed a significant decrease in serum IgG concentrations in patients following efgartigimod treatment, which stabilized at follow‐up. This trend parallels changes in cerebrospinal fluid (CSF) NMDA receptor antibody titers. The correlation between IgG concentrations and antibody titers aligns with previous findings in myasthenia gravis patients treated with efgartigimod, suggesting that it may reduce pathogenic NMDA receptor antibody titers by lowering IgG concentrations [12]. However, while plasma antibody titers showed a downward trend post‐treatment, the differences were not statistically significant. This may be attributed to the lower sensitivity of commonly used cell‐based assays (CBA) with serum compared to fixed‐cell assays in CSF, as well as the small sample size of our study [34]. To further investigate the relationship between efgartigimod use and favorable outcomes, we performed a multivariate regression analysis. Although treatment with efgartigimod showed an apparent association with good prognosis, the result did not reach statistical significance (OR 6.35, 95% CI 0.77–102.80, *p* = 0.116), likely owing to the limited sample size. Larger studies—including subtype‐specific analyses—are needed to more accurately evaluate the association between efgartigimod therapy and favorable outcomes.

A recent clinical study indicated that patients with anti‐NMDAR encephalitis experienced persistent cognitive deficits after the acute phase, although these deficits gradually improved over time [35]. Notably, nearly half of the control group patients (*n* = 8) exhibited significant cognitive deficits at follow‐up, while significantly fewer patients in the efgartigimod group (*n* = 2) did, suggesting that efgartigimod may enhance cognitive function more effectively than other treatments.

The number of patients treated with rituximab in the efgartigimod group was lower than in the control group because some individuals showed significant improvement after receiving efgartigimod. Rituximab alleviates the symptoms of anti‐NMDAR encephalitis by targeting and eliminating B cells, which reduces the abnormal autoimmune response. However, its onset of action may be slower than that of efgartigimod, which rapidly decreases pathogenic antibodies. The therapeutic effects of rituximab may become more apparent during long‐term follow‐up. These findings also support adding efgartigimod into first‐line immunotherapy. Early initiation of efgartigimod may accelerate recovery and reduce the need for more invasive procedures. Therefore, in clinical practice, the following immunotherapy procedures may lead to better prognosis: (1) initiate high‐dose steroids on Day 0; (2) if no measurable improvement within Day 3–5, add efgartigimod once a week for 2 weeks; (3) consider IVIG or PE/IA only if the response remains inadequate [36, 37, 38, 39].

In this study, no patients discontinued efgartigimod due to TEAEs. In both the efgartigimod and control groups, one patient experienced a moderate drop in WBC count. In the efgartigimod group, this occurred following the first treatment and was associated with fever and a urinary tract infection; in the control group, it occurred after the first rituximab treatment and was linked to fever and a lung infection. The primary TEAEs associated with efgartigimod are mild to moderate headaches and infections [8]. For instance, in studies treating pemphigus vulgaris and foliaceus, the main TEAEs observed included headaches, diarrhea, and infections [21]. Similarly, another study on myasthenia gravis noted headaches and infections as the primary TEAEs [10]. Similar findings were reported in the pemphigus vulgaris/foliaceus study and in the CIDP study [15]. Our findings align with these studies, extend these observations to anti‐NMDAR encephalitis, suggesting that efgartigimod confers clinical benefit without adding significant safety burden. While the observed infections may relate to concomitant corticosteroid and immunosuppressant use, a connection to efgartigimod cannot be ruled out. Therefore, monitoring WBC counts and potential secondary infections is essential when combining efgartigimod with corticosteroids.

This study has limitations, including a small sample size and its retrospective nature. Nevertheless, our findings support the safety and efficacy of efgartigimod in treating NMDA receptor encephalitis. We did not measure efgartigimod concentrations in cerebrospinal fluid (CSF) and blood, leaving uncertain whether it can cross the blood–brain barrier and exert direct effects within the CNS. Based on CSF antibody titers, we hypothesize that efgartigimod can reduce pathogenic antibody concentrations in the CSF. However, it remains unclear whether this clearance is due to efgartigimod crossing the blood–brain barrier or a secondary effect resulting from a dynamic equilibrium due to reduced blood antibody levels. Additionally, the optimal dosage and administration of efgartigimod in patients with NMDAR encephalitis warrant further investigation. Therefore, additional research is essential to elucidate efgartigimod's mechanism of action, determine optimal usage and dosage, and compare its efficacy with other treatments.

## 5 Conclusions

In conclusion, efgartigimod treatment for NMDA receptor encephalitis was associated with no apparent adverse effects and accelerated improvements in neurological dysfunction and clinical symptoms, particularly cognitive function. This not only alleviates the burden on patients' families but also optimizes healthcare resources. Efgartigimod represents a promising novel therapeutic option for anti‐NMDAR encephalitis.

## Author Contributions

All the authors had full access to all data used in this study and take responsibility for the integrity and accuracy of the data analysis. Z.B. and B.Z. wrote and revised the manuscript. W.Q., F.P., Z.L., and B.Z. designed and supervised the nonclinical studies, and Z.B., H.C., H.W., and P.L. acquired the nonclinical data. Z.B., H.C., H.W., F.Z., and P.L. wrote the clinical study protocol and supervised the study. Z.B., H.C., H.W., F.Z., and P.L. analyzed and interpreted the data.

## Ethics Statement

This study was approved by the Institutional Review Board of The Third Affiliated Hospital of Sun Yat‐sen University, China.

## Consent

All participants or their legal representatives provided written informed consent.

## Conflicts of Interest

The authors declare no conflicts of interest.

## Supporting information


**Table S1:** Univariate and multivariate logistic regression analysis of treatment group.

**Table S2:** Immunotherapies applacation before efgartigimod treatment.

## Data Availability

The data supporting the findings of this study are available on request from the corresponding author.
